# Sex-dependent genetic effects on immune responses to a parasitic nematode

**DOI:** 10.1186/1471-2164-15-193

**Published:** 2014-03-14

**Authors:** Kelly S Hayes, Reinmar Hager, Richard K Grencis

**Affiliations:** Faculty of Life Sciences, University of Manchester, Manchester, M13 9PT UK

**Keywords:** Sex-dependent effect, Parasite, Gut, *Trichuris muris*, QTL, BXD, Mice

## Abstract

**Background:**

Many disease aetiologies have sex specific effects, which have important implications for disease management. It is now becoming increasingly evident that such effects are the result of the differential expression of autosomal genes rather than sex-specific genes. Such sex-specific variation in the response to *Trichuris muris*, a murine parasitic nematode infection and model for the human parasitic nematode *T. trichiura*, has been well documented, however, the underlying genetic causes of these differences have been largely neglected. We used the BXD mouse set of recombinant inbred strains to identify sex-specific loci that contribute to immune phenotypes in *T. muris* infection.

**Results:**

Response phenotypes to *T. muris* infection were found to be highly variable between different lines of BXD mice. A significant QTL on chromosome 5 (*TM5*) associated with IFN-γ production was found in male mice but not in female mice. This QTL was in the same location as a suggestive QTL for TNF-α and IL-6 production in male mice suggesting a common control of these pro-inflammatory cytokines. A second QTL was identified on chromosome 4 (*TM4*) affecting worm burden in both male and female cohorts. We have identified several genes as potential candidates for modifying responses to *T. muris* infection.

**Conclusions:**

We have used the largest mammalian genetic model system, the BXD mouse population, to identify candidate genes with sex-specific effects in immune responses to *T. muris* infection. Some of these genes may be differentially expressed in male and female mice leading to the difference in immune response between the sexes reported in previous studies. Our study further highlights the importance of considering sex as an important factor in investigations of immune response at the genome-wide level, in particular the bias that can be introduced when generalizing results obtained from only one sex or a mixed sex population. Rather, analyses of interaction effects between sex and genotype should be part of future studies.

**Electronic supplementary material:**

The online version of this article (doi:10.1186/1471-2164-15-193) contains supplementary material, which is available to authorized users.

## Background

Many human diseases differ in prevalence, course and severity between males and females [1, 2]. This sex bias is most obvious in human autoimmune conditions [3] that predominantly affect women. Additionally, many other diseases such as cardiovascular disease, osteoporosis and Alzheimer’s also have differing effects in males and females [1–3]. Males and females differ genetically by only a few genes located on sex-specific chromosomes, which are unlikely to account for the vast range of phenotypic differences. It has become increasingly evident that these sex-specific phenotypes are due to differentially expressed genes present in both sexes [4–6]. Such genotype by sex interactions have been observed in a number of organisms, from insects [7] to mammals [8]. Further, sexually dimorphic gene expression patterns are often tissue specific suggesting that different regulatory interactions might control gene expression in different tissues. This can involve genes exclusively expressed in one sex or genes expressed predominantly in one sex. The latter are often referred to as male or female biased genes, where male biased genes are generally more functionally diverse than female biased genes [9].

Over three billion people worldwide, mainly in developing countries, are thought to be susceptible to stable transmission of one or more of the four geohelminth species, *Ascaris lumbricoides*, *Trichuris trichiura*, *Ancylostoma duodenale* and *Necator americanus*[10]. *T. trichiura*, the human whipworm, is a key parasitic nematode that is known to cause considerable morbidity. This nematode has been the focus of much research (reviewed in [11]) using *T. muris,* the mouse whipworm*,* as a well-established model system. The immune response to this infection in mice is very well characterised and there is a distinct polarisation of immune response in resistant and susceptible strains of mouse [12, 13]. Resistant animals produce high levels of interleukin 13 (IL-13) and associated T helper type 2 (Th2) cytokines in response to infection, which is essential for parasite expulsion. In contrast, a susceptible animal produces high amounts of interferon γ (IFN-γ) and associated Th1 cytokines that leads to chronic infection. Importantly, the term resistance in this model is used when expulsion of the worms occurs before they become sexually mature and in both resistant and susceptible animals, *T. muris* establishes within the intestine. Resistance to *T. muris* has been demonstrated to have a strong genetic component with both H2 (major histocompatibility complex) linked genes and background genes influencing immunity [14, 15].

A known sex bias exists in immunity to *T. muris* infection [16, 17] whereby female mice are known to mount a stronger Th2 response to infection and are thus more resistant. This phenotype is more readily seen in genetically deficient animals such as the IL-4 KO mouse, although a weaker effect is still detectable in wild type mice [18]. Such sex-specific immune responses are also known in other models of helminth infection [19, 20]. Thus, while differences between sexes in immunity have been well documented, few studies have attempted to identify the underlying genetic variants that may cause such sex-specific responses. To date, a genome-wide analysis of genetic variants whose effects on immune response differ between the sexes has not been conducted in experimental populations.

In this study, we focus on sex-specific genetic effects on immune response phenotypes to *T. muris* in a population of BXD recombinant inbred (RI) mice. This reference panel consists of experimentally tractable mouse lines capturing a large amount of naturally occurring genetic variation and is ideally suited to integrate and analyse massive phenotypic data sets [21, 22] thus providing a valuable tool to identify loci that contribute to immune phenotypes in *T. muris* infection. To determine the heritable differences in immune phenotypes to *T. muris*, we profiled parasite burden and infection-induced cytokine responses in peripheral blood in 20 BXD lines and both parental strains, C57Bl/6J and DBA/2J. Our analysis concentrated on cytokines that are reliably measurable in serum as a reflection of inflammatory/immune events occurring in the infected animal. We used interval mapping and gene-mining tools in GeneNetwork (GN) to identify novel candidate loci involved in promoting immunity without bias to any particular subset of genes. We mapped a significant QTL to an interval on chromosome 5 (*TM5*) and an additional suggestive QTL to an interval on chromosome 4 (*TM4*). *TM5* was male-biased suggesting that expression of a gene or multiple genes within this region are differentially expressed in male and female mice. This has important implications not only for highlighting new genes important in immunity to *T. muris* and by extension, *T. trichiura*, but also provides a mechanistic basis for the known sex bias in infection.

## Results

### Response phenotypes to *T. muris*are highly variable

**Figure 1:**
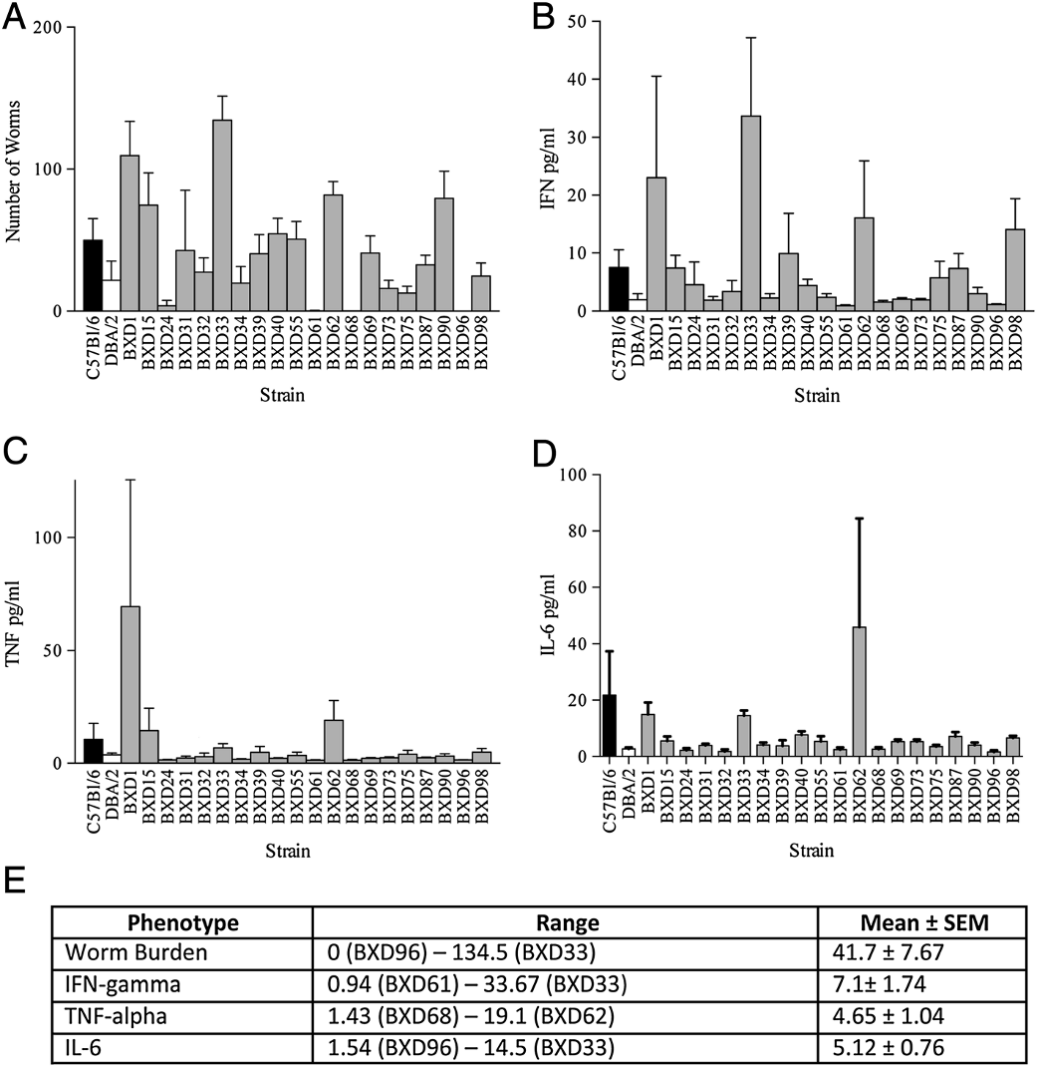
**Immune response phenotypes to** ***T. muris*** **.** Mean ± SEM of immune response phenotypes to *T. muris* **(A)** worm burden, **(B)** serum IFN-γ, **(C)** serum TNF-α and **(D)** serum IL-6 in BXD RI lines (grey bars) and the parental strains C57BL/6 (black bars) and DBA/2 (white bars). **(E)** Summary of range and mean ± SEM of response phenotypes.

The four measured immune phenotypes, worm burden, IFN-γ, Tumour Necrosis Factor α (TNF-α) and IL-6 all showed considerable variation across genotypes (Figure 1). Interestingly, the parental measurements did not represent the spread of the offspring results (Figure 1) and although there were no significant differences between DBA/2 and C57BL/6 mice, there were significant differences between the BXD RI lines (Additional file 1: Table S1). We detected a significant line effect on worm burden (ANOVA, F_21,333_ = 6.681, p < 0.0001), IFN-γ (ANOVA F_21,333_ = 2.289, p < 0.001), TNF-α (ANOVA, F_21,333_ = 1.660, p = 0.03) and IL-6 (ANOVA, F_21,330_ = 5.119, p < 0.0001).

Heritability for each phenotype was calculated by ANOVA with line as the independent variable using batch/age corrected values of our phenotypes. In BXD mice, heritabilities of worm burden by rank (no worms, low numbers, medium numbers and high numbers), IFN-γ, TNF-α and IL-6 were 42%, 22%, 14% and 18%, respectively.

### Mapping immune phenotypes

**Figure 2:**
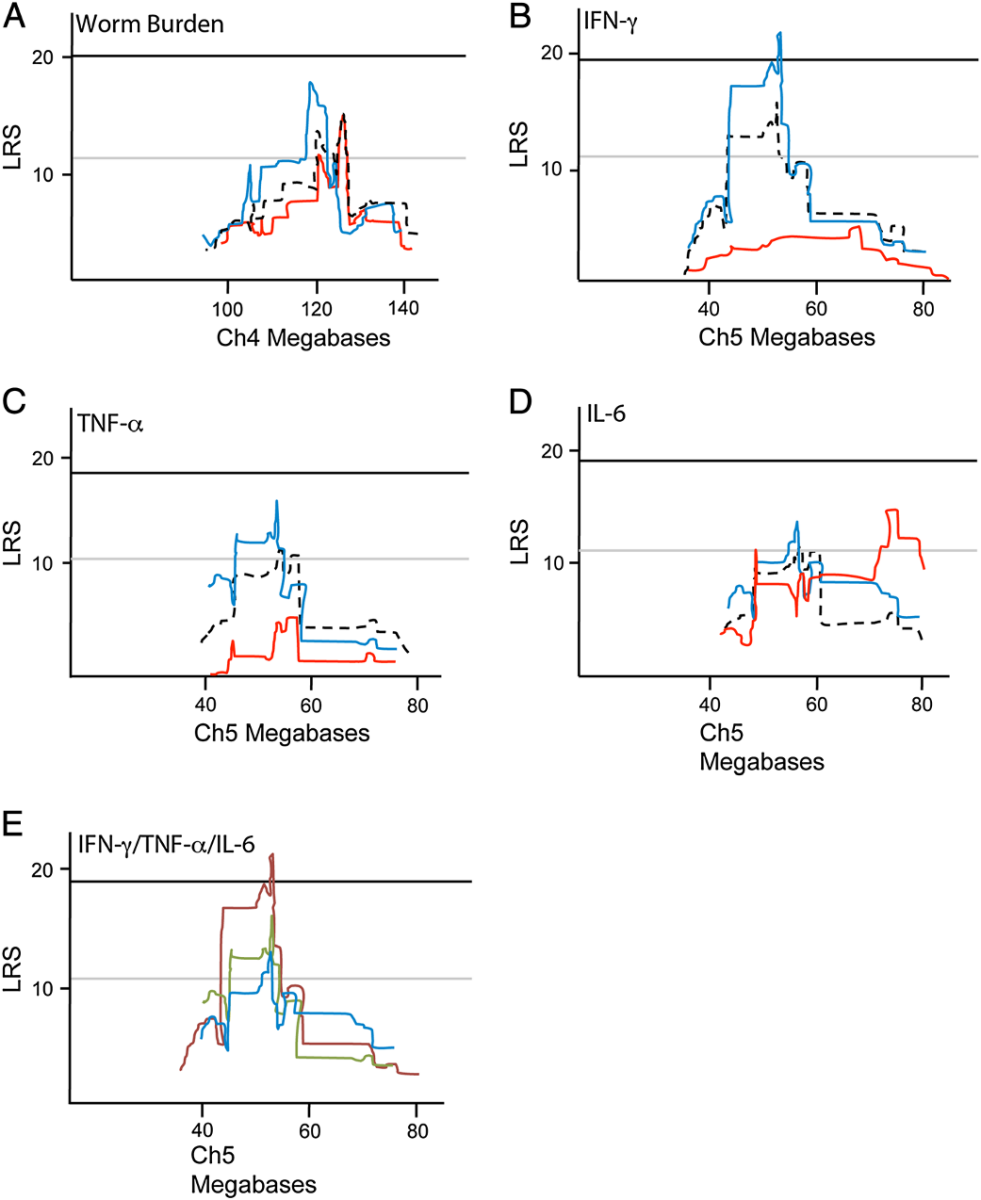
**Interval maps for immune response phenotypes in male, female and combined male and female cohorts.** QTL for male (blue line) and female (red line) as compared to both sexes together (broken black line) for **(A)** worm burden (Ch4), **(B)** IFN-γ, **(C)** TNF-α, **(D)** IL-6 (all Ch5) response phenotypes. **(E)** Overlap of male QTL for INF-γ (red line), TNF-α (green line) and IL-6 (blue line). Upper dark line on maps show significant LRS scores whilst lower lines show suggestive LRS scores.

In order to map variation related to worm burden and the levels of cytokines that were produced in response to infection, rather than possible confounding covariates, we used General Linear Models (GLMs) to remove all covariance associated with differences in age, batch of eggs used for infection, and maternal genotype. We found significant effects of age on worm burden (GLM F_34,318_ = 4.259, p < 0.001), IFN-γ (GLM F_34,314_ = 2.255, p < 0.001) and TNF-α (GLM F_34,314_ = 7.532, p < 0.001). Further, sex had a significant effect on worm burden (GLM F_1,351_ = 3.473, p = 0.063) and IFN-γ (GLM F_1,347_ = 4.269, p = 0.040) but not TNF-α or IL-6. Maternal genotype had significant effects on worm burden (GLM F_1,351_ = 23.695, p < 0.001) and IFN-γ (GLM F_1,347_ = 8.345, p = 0.004) while batch had significant effects on worm burden (GLM F_7,345_ = 3.379, p = 0.002). Residuals of these models were used to compute adjusted immune phenotypes. First, we mapped adjusted worm burden for the entire population and found a suggestive QTL, *TM4*, on Ch4 at 120.5-126.5 Mb (LRS = 13.1). An additional suggestive QTL, *TM5*, was found on Ch5 at 45–55 MB for all adjusted IFN-γ (LRS = 11.0), TNF-α (LRS = 11.8) and IL-6 (LRS = 11.2) (Additional file 2: Figure S1). To investigate sex-specific effects, the data was divided into male and female cohorts and mapped for adjusted worm burden, IFN-γ, TNF-α and IL-6 (Additional file 3: Figure S2 and Additional file 4: Figure S3). As before, there were no significant differences between the parent strains in any phenotype (males or female cohorts) though there were significant differences between lines (Additional file 5: Figure S4 and Additional file 6: Figure S5 and Additional file 7: Table S2). Mapping only male animals significantly improved the strength of linkage to both *TM4* (LRS = 18.4) and *TM5* (LRS = 22.6) (Figure 2A-D). It was immediately apparent from over-laying the QTL maps from male and female data that worm burden maps similarly for both sexes whereas for IFN-γ, TNF-α and IL-6, male data is most strongly linked to *TM5*. Additionally, when the QTL maps for IFN-γ, TNF-α and IL-6 were overlaid, the *TM5* QTL peaks were all in the same position on Ch5, though it only reached significance with the IFN-γ phenotype (Figure 2E).

Both loci were linked to susceptibility. The DBA/2 (D2) allele at *TM4* was associated with a 2-fold increase in worm burden when compared with strains having a C57BL/6 (B6) allele (t_16_ = 2.278, p = 0.04), while a B6 allele at *TM5* was associated with a 2-fold increase in IFN-γ production (t_19_ = 2.421, p = 0.02).

### Candidate gene analysis

Candidate genes were selected using the QTLminer module of GN that ranks genes by whether the parent strains have non-synonymous SNPs (nsSNPs) or indels, whether the candidates are expressed in tissues of interest and whether their expression is modulated by cis-eQTL. For each of these four categories a score is assigned to candidates, with a maximum of 4. As GN does not have a gene expression database for the caecum or large intestine, we instead used mRNA expression from naïve T helper cells and leucocytes [23]. However, as worm burden is known to be dependent on gut function [24, 25] we did not reject genes as candidates if they were not expressed in these target cells, provided they had a known biological relevance to the gut. Of the 93 genes within *TM4*, 16 genes scored 3 or higher out of the possible 4. This was further reduced to ten genes (Table 1) by choosing only candidates located within the QTL for both male and female cohorts (120.5 MB – 126.5 MB), which are therefore likely to be controlled similarly in both males and females.

**Table 1:** **Candidate genes in** ***TM4***

Gene symbol	Description	Mb location	LRS	SNP region
Cap1	CAP, adenylate cyclase-associated protein 1	122.53	16.81*	3′ UTR, exon 2, 8 and 10
Mycbp	c-myc binding protein	123.58	16.81*	3′ UTR
Zmpste24	Zinc metallopeptidase, ste24 homolog	120.73	18.45*	3′ UTR, exon 1 and 2
Ppt1	Palmitoyl-protein thioesterase 1	122.51	16.81*	3′ UTR and exon 6
Mtf1	Metal response element binding transcription factor 1	124.47	16.81*	3′ UTR, 5′ UTR, exon 11
Macf1	Microtubule-actin crosslinking factor 1	123.02	16.81*	Exons 1, 9, 10, 19, 20, 35–40, 43–45, 47–48
Zfp69	Zinc finger protein 69	120.6	15.58*	5′ UTR, exons 3, 4 and 6
Smap2	Stromal membrane-associated GTPase-activating protein 2	120.64	15.58*	5′ UTR
Inpp5b	Inositol polyphosphate-5-phosphatase B	124.41	16.81*	Exon 1, 2, 3 and 8
Rspo1	R-spondin homolog	124.66	9.52	Intronic only

*Suggestive LRS = 12.23.

Similarly, QTLminer was used to identify candidate genes in *TM5*. Again, T helper cells and leucocyte mRNA expression databases were used, as these are the principal cells that would be producing IFN-γ in the serum. Of the 44 genes within this QTL region, only 19 genes had nsSNPs between B6 and D2. Of these 19, seven genes (Table 2) additionally had indels, high expression in target tissues and/or evidence of cis regulation in target tissues.

**Table 2:** **Candidate genes in** ***TM5***

Gene symbol	Description	Mb location	LRS	SNP region
Pi4k2b	Phospohatidylinositol 4-kinase type 2 beta	53.13	18.61*	Exons 1, 2 and 7
Lap3	Leucine aminopeptidase 2	45.88	17.45*	Exons 1 and 5
Tapt1	Transmembrane anterior posterior transformation 1	44.56	18.45*	3′ UTR
Sepsecs	Sep (O-phosphoserine) tRNA:Sec (selenocysteine tRNA synthase)	53.03	18.61*	3′ UTR, exon 7
Lcorl	Ligand dependent nuclear receptor corepressor-like	46.12	17.45*	3′ UTR, exons 1 and 4
Anapc4	Anaphase promoting complex subunit 4	53.22	18.61*	Exons 1 and 4
Slit2	Slit homolog 2 (Drosophila)	48.37	17.45*	3′ UTR, 5′ UTR, exons 9, 17, 20, 26 and 32
SOD3	Superoxide dismutase 3	52.75	18.61*	3′ UTR

*Suggestive LRS = 11.89.

### Epistasis

**Figure 3:**
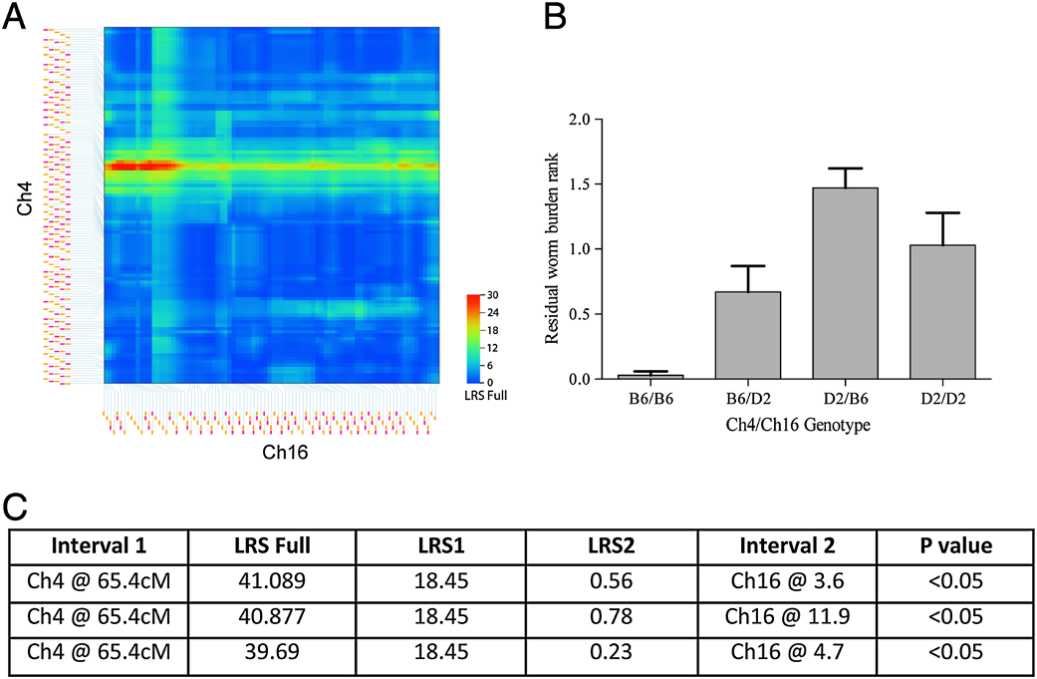
**Pair-scan correlation between two chromosomes for worm burden.** Pair scan analysis demonstrates significant interactions between *TM4* and *TM16* for the worm burden phenotype. **(A)** Enlargement of a significant interaction of *TM4* and *TM16* from the pair-scan analysis. **(B)** Histogram illustrating the effect of adjusted worm burden of carrying either the maternal (B6) or paternal (D2) or both alleles at the *TM4* and *TM16* intervals. **(C)** LRS scores of *TM4* and *TM16*. *p < 0.05.

We next investigated epistatic interactions (i.e. gene by gene interactions) and found a significant interaction between *TM4* and a locus on Ch16, *TM16* (Full model LRS = 39.03, p < 0.05) (Figure 3A and C). Those strains with B6 alleles at both QTL have significantly reduced worm burdens (Figure 3B). The genetic correlation between markers at these two loci was not significant (r = 0.051, p = 0.8), thereby excluding the possibility of non-syntenic association [26]. No epistatic interactions with *TM5* were found.

**Figure 4:**
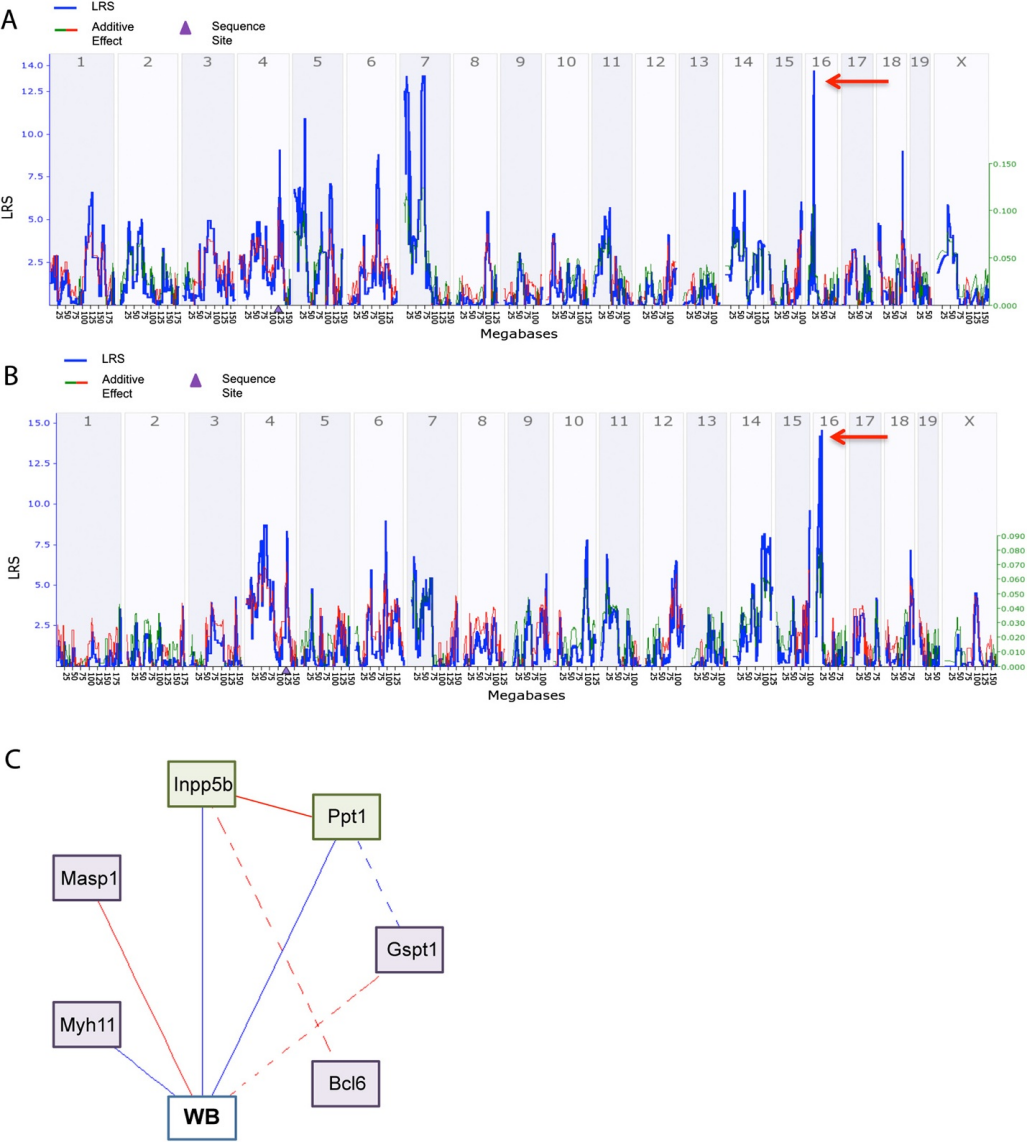
**Correlations and networks for genes in** ***TM4*** **and** ***TM16*** **. (A)** Interval map for *Ppt1* gene expression. This *TM4* gene modulates gene expression in the *TM16* region (red arrow). **(B)** Interval map for the *TM4* gene *Innpb5* that also modulates gene expression in the *TM16* region (red arrow). **(C)** Hypothetical gene network modulating worm burden based on gene correlations between *TM4* and *TM16*. Solid blue lines represent correlations between -0.7 and -0.5, broken blue lines between -1.0 and -0.7. Solid and broken red lines represent the equivalent positive correlations. Phenotypes are in white boxes; *TM4* genes are in green boxes whilst *TM16* genes are in purple boxes. p ≤ 0.01 in all cases.

To further investigate the interaction effects of *TM4* and *TM16*, we looked for correlations in mRNA expression in T helper cells and leucocytes across BXD lines in genes contained within both intervals using GN databases. *TM16* contains 252 genes, of which 58 had nsSNPs between B6 and D2. Again, QTLminer was used to rank candidate genes based on nsSNPs between the parental strains, indels, expression in target tissues or cells and evidence of cis-eQTLs. Of the 252 genes, 16 genes scored highly, of which only seven correlated with expression of genes in *TM4*. An additional two genes were included (Table 3) that did not score highly but were nonetheless considered to be biologically relevant; *Masp1* and *Clcn2*. Interestingly, Ch4 genes *Ppt1* and *Inpp5b* map as trans-QTL to Ch16 (Figure 4A and B respectively), at the locus that is in epistatic interaction with *TM4*. To summarize the interactions of genes in *TM4* and *TM16*, we have constructed a model based on the expression and correlation of these genes with the worm burden phenotype (Figure 4C).

**Table 3:** **Candidate genes within the interactions locus** ***TM16***

Gene	Description	Mb location	SNP region
Abcc1	ATP-binding cassette, sub-family C (CFTR/MRP), member1	14.36	3′ UTR and multiple exons (13)
Mkl2	MKL/Myocardin-like 2	13.35	Exons 9, 10, 11, 12, 13 and 15
Gspt1	G1 to S phase transition 1	11.21	3′ UTR, exons 1 and 2
Nde1	Nuclear distribution gene E homolog 1	14.16	Exons 1-4
Dexi	Dexamethasone-induced transcript	10.53	3′ UTR
Bcl6	B-cell leukaemia/lymphoma 6	23.96	Exon 2
Senp2	SUMO/Sentrin specific peptidase 2	22.00	Exon 1
Myh11	Myosin, heavy polypeptide 11, smooth muscle	14.19	Multiple exons (11)
CIIta	Class II transactivator	10.48	3′ UTR, exons 9, 10 and 11
Masp1	Mannan-binding lectin serine peptidase 1	23.45	Exon 16
Clcn2	Chloride channel 2	20.70	Exons 1 and 2

### Interactions between Worm Burden and IFN-γ phenotypes

**Figure 5:**
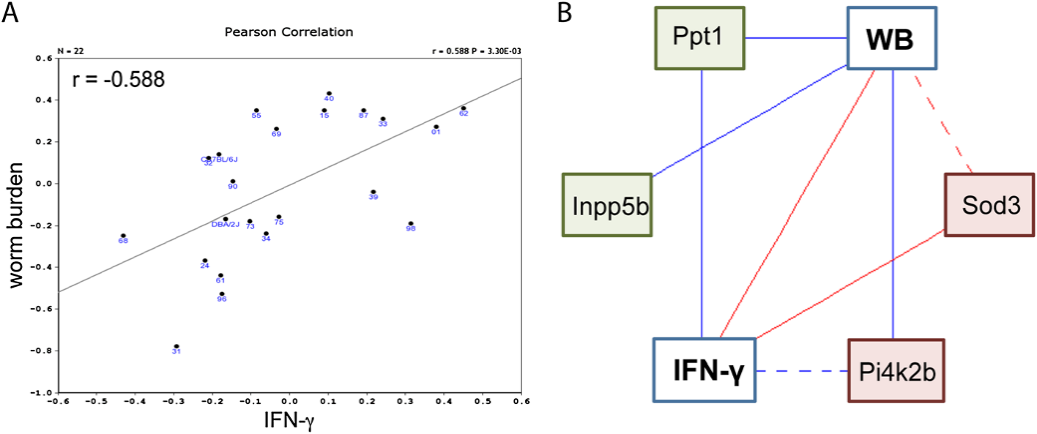
**Correlation and networks for worm burden and IFN-γ phenotype genes. (A)** Spearman rank correlation (in box) of worm burden with IFN-γ response phenotype. **(B)** Hypothetical gene network modulating worm burden based on gene correlations between genes in *TM4* and *TM5*. Solid blue lines represent correlations between -0.7 and -0.5, broken blue lines between -1.0 and -0.7. Solid and broken red lines represent the equivalent positive correlations. Phenotypes are in white boxes, *TM4* genes are in green boxes and *TM5* genes are in red boxes. p ≤ 0.01 in all cases.

Our worm burden phenotype and IFN-γ phenotypes positively correlate with each other (Figure 5A) as expected; it is known that high worm burdens at day 35 post infection (p.i.) are associated with an increased production of IFN-γ. Correlations between the expression of genes in *TM4* and *TM5* in T helper cell and leucocytes were identified on GN and have been summarised in a network graph (Figure 5B).

## Discussion

Sex bias in many diseases has been well documented, particularly with autoimmune diseases, and is attributable in part to the different immune responses that male and females are able to mount. Such sex-dependent effects are potentially caused by differential expression of autosomal genes [8, 9], yet the underlying genetics of this sex-dependence has hitherto been poorly explored. However, if we wish to develop effective interventions we need to understand the genetics of sex-dependent immune responses.

In an effort to address the paucity of data on genetic architecture underlying sex-specific immune response at a genome-wide level we identified loci *TM4* and *TM5* associated with susceptibility parameters of infection: worm burden and the production of IFN-γ, a Th1 cytokine. Moreover, suggestive QTL can be found in exactly the same position as *TM5* when looking at two other susceptibility associated cytokines, IL-6 and TNF-α, suggesting a common control of all these pro-inflammatory cytokines. *TM5* was only apparent when analysing male data whereas *TM4*, associated with worm burden, was apparent in both male and female cohorts. This would suggest that worm burden is controlled similarly in both sexes, whereas the production of the pro-inflammatory cytokines in response to *T. muris* is differentially controlled in male and female mice, in particular given that this QTL is not located on chromosome X or Y. This phenomenon is also seen in other disease systems, such as coronary heart disease and kidney diseases [3] highlighting the importance of sex on disease outcome. Another recent QTL study utilising the *T. muris* system [27] has been conducted using an intercross approach between AKR and BALB/c mice with different parameters of infection considered (absolute worm burden and antibody response). Thus, our study here has identified different QTL associated with sex-dependent immune responses, rather than QTL associated with colitis as in the intercross study [27].

### Parasite niche in the host

*T. muris* is a parasite that lives in close association with its host, embedding into the caecal epithelium and therefore causing damage to the integrity of the gut. It is further known that the intestinal epithelium plays a key role in expulsion [24], as resistant animals have a faster rate of epithelial turnover than susceptible animals. Furthermore, increasing the turnover rate in susceptible animals enables expulsion. This process will require a fine balance between apoptosis and proliferation within the gut. Our study has identified several genes from three different chromosomes that play a role in epithelial barrier integrity and host responses to damage.

*MacF1* is a member of the Wnt signalling pathway [28], a highly conserved signal transduction cascade that is known to play a role in gut epithelium homeostasis [29]. Although *Rspo1* does not score highly in the system used to identify candidate genes, it is biologically relevant in the context of *T. muris* infection as it is involved in the proliferative control of intestinal crypt cells, which will be in close association with the parasite, and enhances Wnt/β-catenin signalling in the intestine [30, 31]. Additionally, QTLminer analysis does not include intronic SNPs that may have regulatory functions, which can be found within the *Rspo1* gene. Thus these two genes may be important in *T. muris* infection by regulating the speed of epithelial turnover in the intestine. *Rspo1* has also been shown to ameliorate colitis in mice by improving the mucosal integrity of the gut [32]. *Clcn2* is known to be important in regulating intestinal mucosa barrier function [33] and like *Rspo1*, is important in the recovery of the epithelial barrier in the intestine after damage [34], which will be important in *T. muris* infection. Not only has mucus been shown to be extremely important in protecting against *T. muris* infection [35] but also that *T. muris* worms secrete specific proteins to break down the mucus barrier [36]. As turnover is finely balanced with apoptosis [37] in the gut, and apoptosis has been shown to be important during *T. muris* infection [25], the candidate gene *Ppt1* is of particular interest because it is known to modulate TNF-α induced apoptosis [38]. Accelerated turnover is thought to dislodge *T. muris* from its niche, pushing it into the lumen of the gut where 'weep and sweep’ mechanisms will then facilitate expulsion. *Myh11* is a contractile protein of smooth muscle and so may be important in the expulsion of *T. muris* worms by peristalsis or by maintaining the integrity of the smooth muscle in the intestine [39].

### Damage to the host and repair

Another aspect of *T. muris* chronic infection is the damage caused by the parasite burrowing through the epithelium. A number of studies on intestinal inflammation have shown a close association of this inflammation with tissue hypoxia. *T. muris* has been shown to share many phenotypic characteristics of Inflammatory Bowel Diseases (IBD) [40] and as damage is sustained during infection, then it is likely that genes in the hypoxia pathway are likely to be important in protecting the host. *SOD3* is an antioxidant enzyme that is known to protect tissues against hypoxic stress [41, 42] and is a primary scavenger of superoxide. It is known that the induction of HIF-1α is regulated closely by oxidant/antioxidant equilibrium involving *SOD3*[43]. Interestingly, HIF-1α has been shown to be upregulated during chronic *T. muris* infection (data not shown). Another hypoxia gene highlighted in this study is *Mtf1*, which induces metallotheionein transactivation [44]. Metallothioneins are a family of small proteins that may have a potential role in IBD [45] and as such may indeed play a role in *T. muris* infection.

### Adaptive immune responses to infection

*T. muris* infection is controlled largely by the production of Th2 cytokines. Several genes involved in T cell signalling have been identified in this study. *MacF1*, as well as being important in the homeostasis of the gut epithelium, may also be important as down stream events of *MacF1* signalling, such as beta-catenin degradation, are implicated in T cell receptor signalling [46, 47]. *Pi4k2b* is associated with components of the T cell receptor [48, 49] and so may also play an important role in *T. muris* infection. *CIIta* is essential for controlling MHCII expression [50] and so is important in Th2 cell recognition of parasite antigens and subsequent activation. A mutation in this protein is known to upregulate IL-33 dependent differentiation of Th2 cells [51], a process that has been shown to be critical in immunity to *T. muris*[52]. *Senp2* is another member of the Wnt signalling pathway, like *MacF1*[53]. Additionally, it can conjugate with PPARγ, a nuclear receptor, affecting the transcription of PPARγ response genes [54]. PPARγ is known to have multiple functions in the immune system [55, 56], including protection against IBD.

The loci we identified in this study are susceptibility loci. Susceptible animals mount a strong Th1 response to infection that then facilitates the survival of this parasite. It is not clearly understood why susceptible animals do not mount a Th2 response to infection. Interestingly, two genes identified in this study are known to be negative regulators of immune responses. *Slit2* inhibits dendritic cell and neutrophil migration [57–59] whereas Bcl6 is known to negatively regulate Th2 immunity [60, 61]. Thus, it may be that susceptible animals mount strong Th1 responses, at least partly via down-regulation of Th2 immunity through such genes.

Finally, the two response phenotypes, IFN-γ and worm burden, measured in this experiment were also found to positively correlate with each other and further correlations between expression levels in *TM4* and *TM5* were identified. This has therefore allowed us to construct hypothetical models of interactions between genes on three different chromosomes based on correlation and expression.

## Conclusions

Sex-dependence is known to play an important role in the prevalence or severity of diseases and it is becoming increasingly apparent that it is variation within the autosomal genome that cause these wide ranging effects. This study further highlights the importance of sex in parasitic infections and identifies several genes that may be differentially regulated and/or expressed in male and female animals. This not only has implications for research, often dominated by single sex studies, but may also lead to the identification of genes important in *T. muris* infection that are not considered solely due to a known role in immune responses, a bias that will discount a myriad of genes that may well be critical.

## Methods

### Mice

C57BL/6JOlaHsd and DBA/2J parent mice were obtained from Harlan and BXD recombinant inbred mice were obtained from the University of Tennessee Health Science Centre, Memphis, TN, USA. All mice were subsequently bred and maintained under specific pathogen-free conditions at the University of Manchester. 353 mice were used in this study (174 males and 179 females) and analysed as groups within lines. Mice in all lines ranged in age from 12–40 weeks and at least 6 males and 6 females from each line were used. All procedures conformed to the requirements of the UK Animals (Scientific Procedures) Act 1986, were subject to local ethical review by the University of Manchester Ethical Review Panel and followed ARRIVE guidelines.

BXD is the largest and best phenotyped genetic model system in mammals, and is derived from two divergent mouse strains (C57**B**l/6 J and **D**BA/2 J, hence **B**X**D**), in which different recombination patterns have been inbred (hence recombinant inbred) in over 100 highly diverse lines, each with a fixed recombination pattern of exactly two possible alleles [21, 22]. These strains incorporate 4–5 million segregating single nucleotide polymorphisms, 500,000 insertions and deletions, and 55,000 copy number variants (1 kb to 100 kb). This is sufficient complexity to model the genetics of human populations used in linkage and genome-wide association studies (GWAS).

### Parasite maintenance and infection

Stock infections of *T. muris* were maintained in susceptible mouse strains and adult worms harvested at day 42 p.i. Adult worms were cultured in RPMI 1640 supplemented with 10% FCS, 2 mM L-glutamine, 100 U/mL penicillin and 100 μg/mL streptomycin (all Invitrogen, UK) and Excretory/Secretory (E/S) antigen and eggs were collected after four hours incubation. *T. muris* E/S was prepared as follows. The E/S was pelleted to remove eggs, concentrated using a Centriprep YM-10 (Amicon, Gloucester, UK) and then dialysed against PBS. Protein concentration was determined by Nanodrop. Eggs were allowed to embryonate for at least six weeks in dH_2_O and infectivity established by worm burden in a susceptible mouse strain. Mice were infected with 150–200 embryonated eggs and worm burdens established at day 35 p.i. Seven separate batches of eggs were used for infections, all from the same passage. Parasite specific IgG1 ELISAs as described in [62] were carried out to confirm infection in all animals.

### Immunophenotyping

Adult worm burdens were assessed at day 35 p.i. via longitudinal dissection of caeca and proximal colons. Serum was taken from infected animals at day 35 p.i. Cytokines IFN-γ, TNF-α and IL-6 were measured as they can be detected in sera and provide an indication of the ongoing immune response to *T. muris*. Commercial Cytometric Bead Array Flex Sets (BD, UK) were used to determine the levels (pg/ml) of IFN-γ, TNF-α and IL-6 in sera according to manufacturer’s instructions. Fluorescence was analysed using a flow cytometer (FACSArray, BD) and cytokine levels were determined using BD CBA software. Linear regression models (GLMs) in SPSS were used to adjust for age, batch and maternal genotype in both worm burden and cytokine levels.

### Genetic analysis

QTL analysis was performed using interval mapping [63] as implemented in the WebQTL module of GN [64]. To identify potential candidate genes we used both information from the UCSC genome browser and GO annotation, and QTL miner in GN [65]. Further, we analysed covariation networks and correlated worm burden and IFN-γ phenotypes with BXD genotype databases (leucocyte mRNA and T cell (helper) mRNA) in GN that contain an extensive collection of previously published and unpublished data from BXD [23].

## Statistical analysis

We used ANOVA and univariate General Linear Models as implemented in SPSS (v20).

## Availability of supporting data

All supporting data available as additional files.

## Electronic supplementary material

Additional file 1: Table S1: Table of immune response phenotypes. Multiple comparisons of one-way ANOVA on worm burden, serum IFN-γ, serum TNF-α and serum IL-6 in BXD RI lines and the parental strains C57BL/6 and DBA/2. (CSV 41 KB)

Additional file 2: Figure S1: Interval maps for immune response phenotypes in combined male and female cohorts. QTL for (A) worm burden (*TM4*), (B) serum IFN-γ, (C) serum TNF-α, (D) serum IL-6 (all *TM5*) response phenotypes. Upper red line on maps show significant LRS scores whilst lower grey line shows suggestive LRS scores. (TIFF 2 MB)

Additional file 3: Figure S2: Interval maps for immune response phenotypes in male cohorts only. QTL for (A) worm burden (*TM4*), (B) serum IFN-γ, (C) serum TNF-α, (D) serum IL-6 (all *TM5*) response phenotypes. Upper red line on maps show significant LRS scores whilst lower grey lines show suggestive LRS scores. (TIFF 2 MB)

Additional file 4: Figure S3: Interval maps for immune response phenotypes in female cohorts only. QTL (A) worm burden (*TM4*), (B) serum IFN-γ, (C) serum TNF-α, (D) serum IL-6 (all *TM5*) response phenotypes. Upper red line on maps show significant LRS scores whilst lower grey lines show suggestive LRS scores. (TIFF 2 MB)

Additional file 5: Figure S4: Male immune response phenotypes to *T. muris*. Mean ± SEM of immune response phenotypes in male mice to *T. muris* (A) worm burden, (B) serum IFN-γ, (C) serum TNF-α and (D) serum IL-6 in BXD RI lines (grey bars) and the parental strains C57BL/6 (black bars) and DBA/2 (white bars). (E) Summary of range and mean ± SEM of response phenotypes. (TIFF 406 KB)

Additional file 6: Figure S5: Female immune response phenotypes to T. muris. Mean ± SEM of immune response phenotypes in female mice to *T. muris* (A) worm burden, (B) serum IFN-γ, (C) serum TNF-α and (D) serum IL-6 in BXD RI lines (grey bars) and the parental strains C57BL/6 (black bars) and DBA/2 (white bars). (E) Summary of range and mean ± SEM of response phenotypes. (TIFF 407 KB)

Additional file 7: Table S2: Table of immune response phenotypes in male and female cohorts. Multiple comparisons of one-way ANOVA on worm burden, serum IFN-γ, serum TNF-α and serum IL-6 in BXD RI lines and the parental strains C57BL/6 and DBA/2 in male or female cohorts. (CSV 82 KB)
