# Supplementary figures and images for: Sex-dependent genetic effects on immune responses to a parasitic nematode
Source: BMC Genomics. 2014 Mar 14;15(1):193. doi: 10.1186/1471-2164-15-193 (PMC4022179; doi:10.1186/1471-2164-15-193)

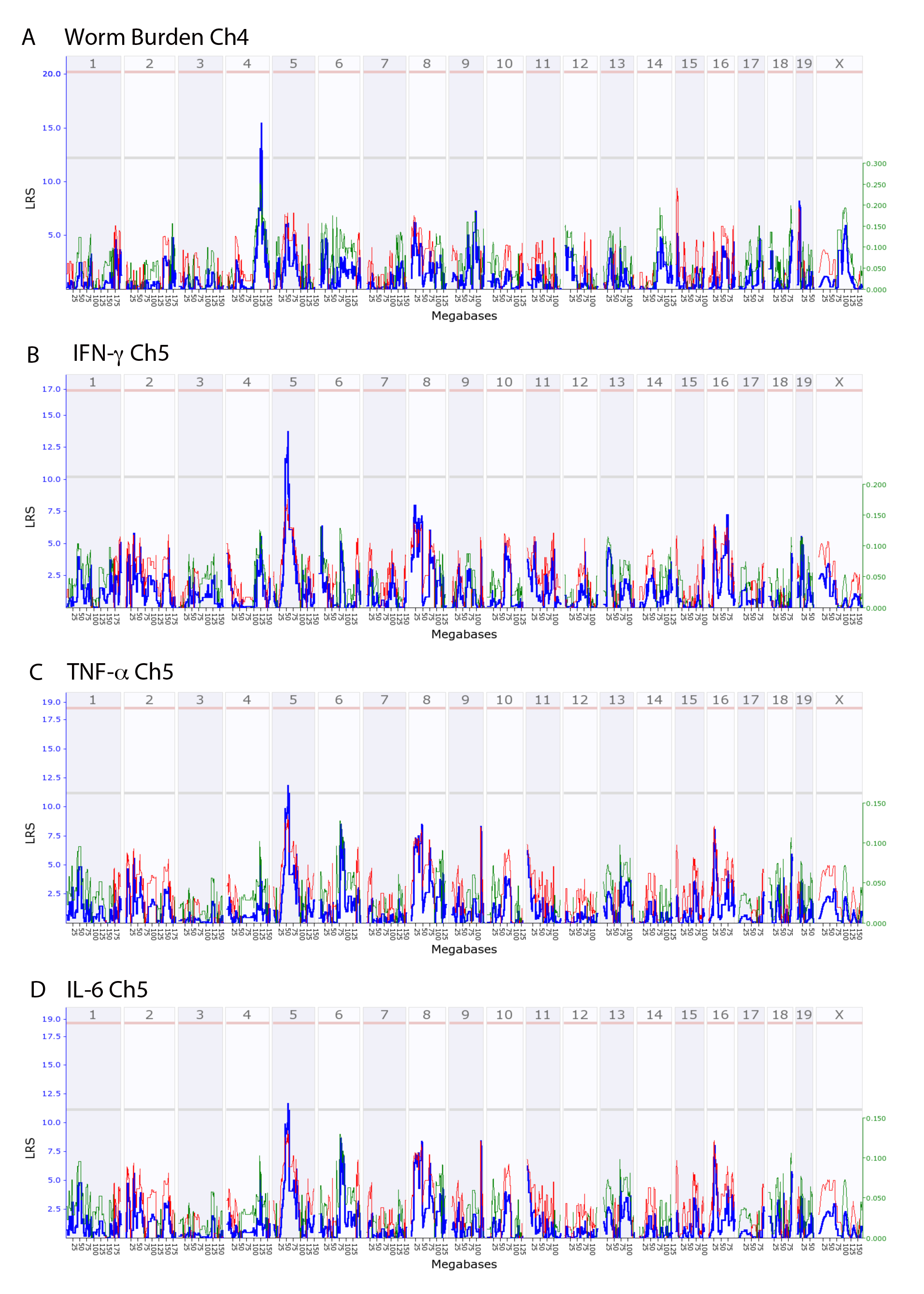

Supplement: Supplementary file 2 — Additional file 2: Figure S1: Interval maps for immune response phenotypes in combined male and female cohorts. QTL for (A) worm burden (TM4), (B) serum IFN-γ, (C) serum TNF-α, (D) serum IL-6 (all TM5) response phenotypes. Upper red line on maps show significant LRS scores whilst lower grey line shows suggestive LRS scores. (TIFF 2 MB) [file 12864_2014_7031_MOESM2_ESM.tiff]

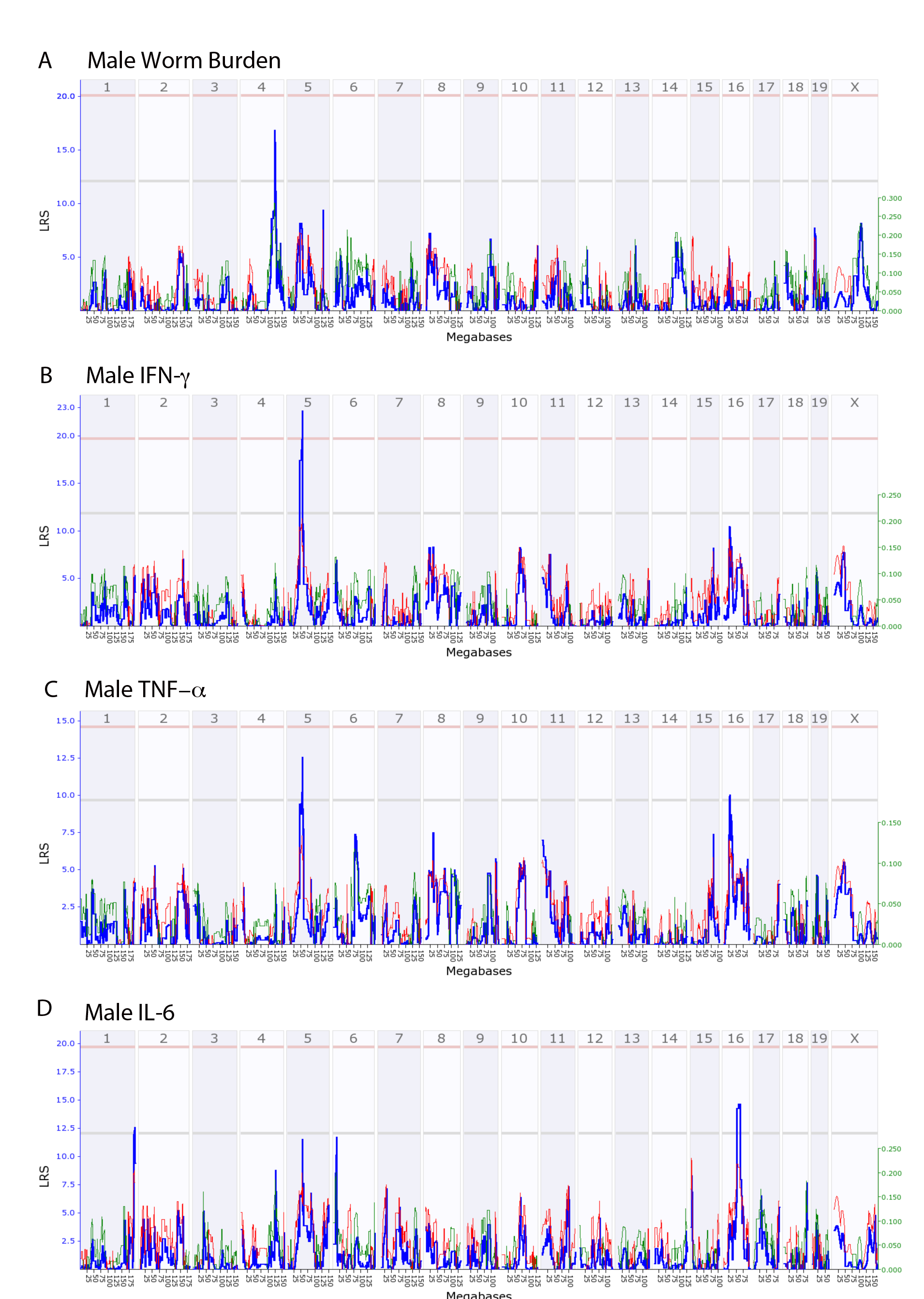

Supplement: Supplementary file 3 — Additional file 3: Figure S2: Interval maps for immune response phenotypes in male cohorts only. QTL for (A) worm burden (TM4), (B) serum IFN-γ, (C) serum TNF-α, (D) serum IL-6 (all TM5) response phenotypes. Upper red line on maps show significant LRS scores whilst lower grey lines show suggestive LRS scores. (TIFF 2 MB) [file 12864_2014_7031_MOESM3_ESM.tiff]

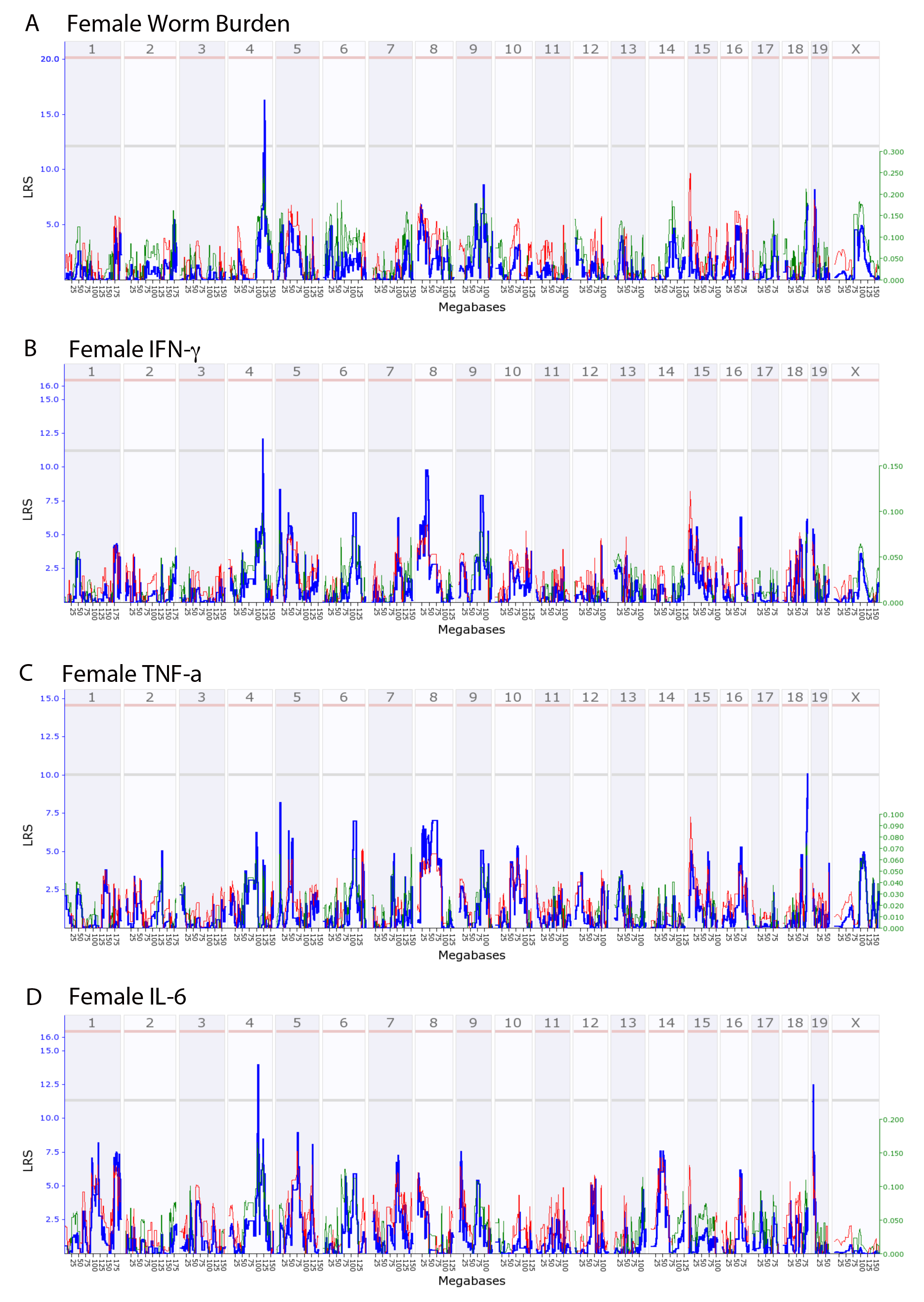

Supplement: Supplementary file 4 — Additional file 4: Figure S3: Interval maps for immune response phenotypes in female cohorts only. QTL (A) worm burden (TM4), (B) serum IFN-γ, (C) serum TNF-α, (D) serum IL-6 (all TM5) response phenotypes. Upper red line on maps show significant LRS scores whilst lower grey lines show suggestive LRS scores. (TIFF 2 MB) [file 12864_2014_7031_MOESM4_ESM.tiff]

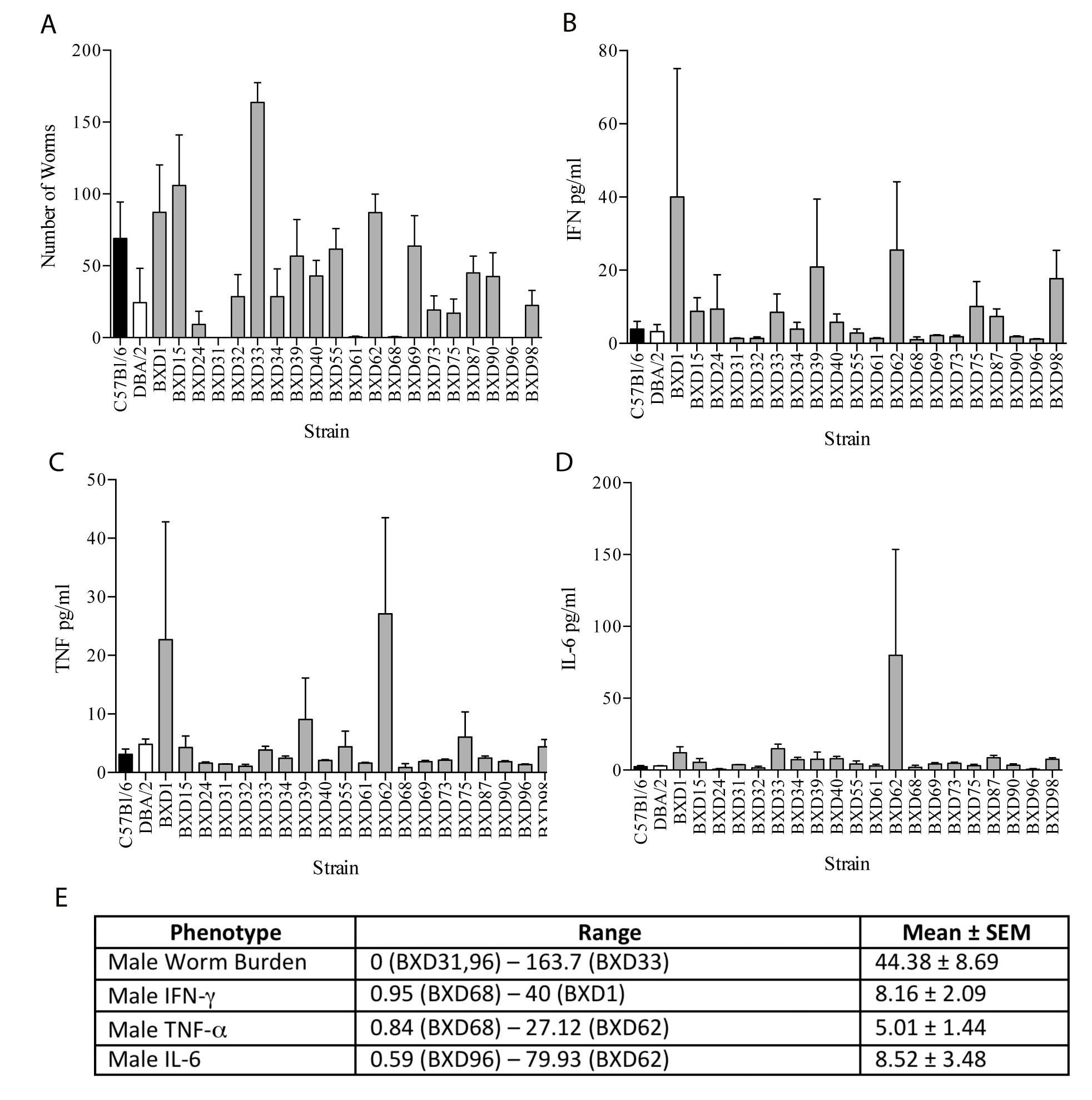

Supplement: Supplementary file 5 — Additional file 5: Figure S4: Male immune response phenotypes to T. muris. Mean ± SEM of immune response phenotypes in male mice to T. muris (A) worm burden, (B) serum IFN-γ, (C) serum TNF-α and (D) serum IL-6 in BXD RI lines (grey bars) and the parental strains C57BL/6 (black bars) and DBA/2 (white bars). (E) Summary of range and mean ± SEM of response phenotypes. (TIFF 406 KB) [file 12864_2014_7031_MOESM5_ESM.tiff]

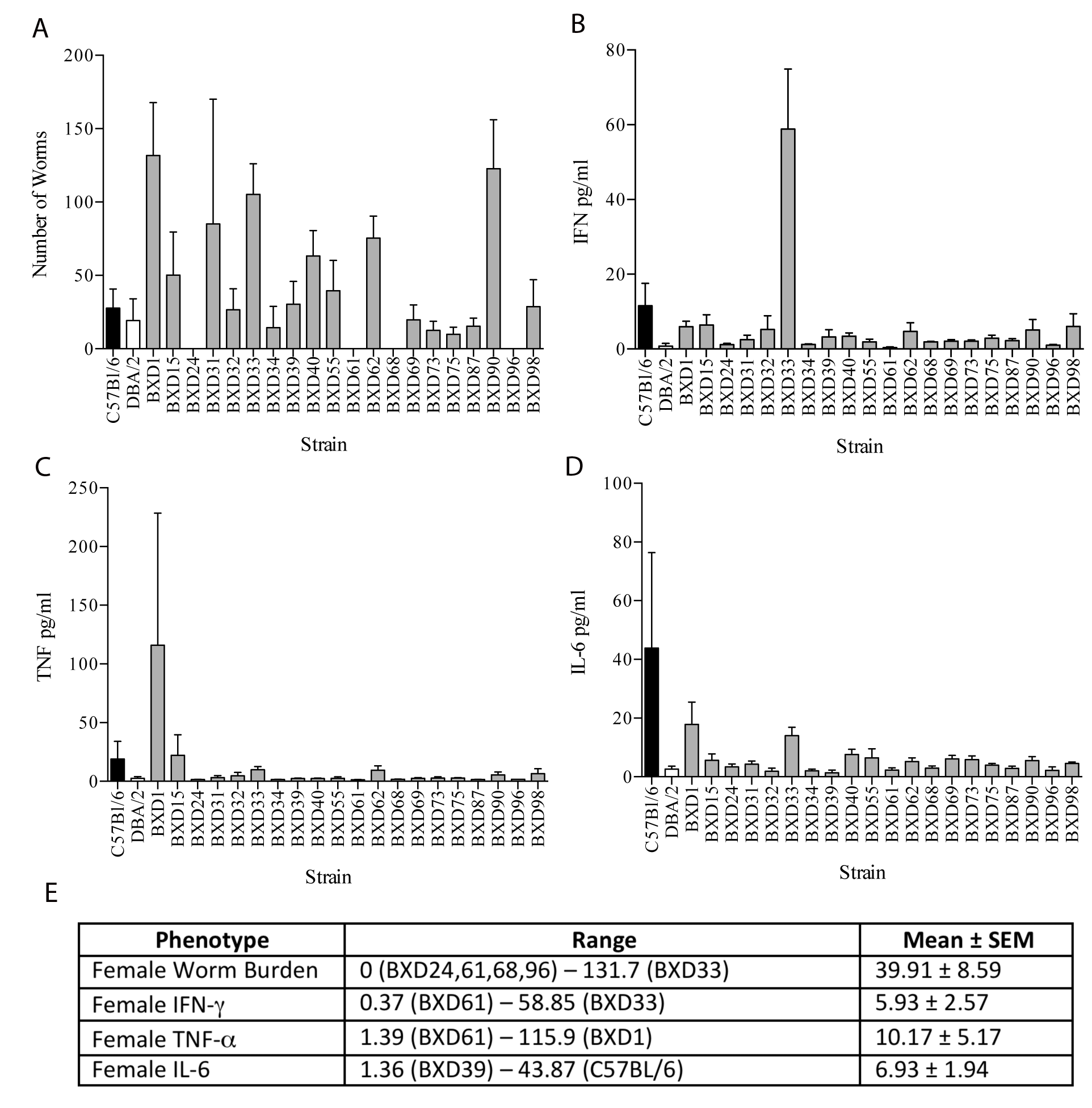

Supplement: Supplementary file 6 — Additional file 6: Figure S5: Female immune response phenotypes to T. muris. Mean ± SEM of immune response phenotypes in female mice to T. muris (A) worm burden, (B) serum IFN-γ, (C) serum TNF-α and (D) serum IL-6 in BXD RI lines (grey bars) and the parental strains C57BL/6 (black bars) and DBA/2 (white bars). (E) Summary of range and mean ± SEM of response phenotypes. (TIFF 407 KB) [file 12864_2014_7031_MOESM6_ESM.tiff]
